# Dynamic Covalent Bonds of Si-OR and Si-OSi Enabled A Stiff Polymer to Heal and Recycle at Room Temperature

**DOI:** 10.3390/ma14102680

**Published:** 2021-05-20

**Authors:** Ping Fan, Can Xue, Xiantai Zhou, Zujin Yang, Hongbing Ji

**Affiliations:** 1 Fine Chemical Industry Research Institute, School of Chemistry, Sun Yat-Sen University, Guangzhou 510275, China; fan18810038712@163.com; 2 Fine Chemical Industry Research Institute, School of Chemical Engineering and Technology, Sun Yat-Sen University, Zhuhai 519082, China; zhouxtai@mail.sysu.edu.cn (X.Z.); yangzj3@mail.sysu.edu.cn (Z.Y.); 3 Maoming Branch, Guangdong Laboratory for Lingnan Modern Agriculture, Maoming 525000, China

**Keywords:** self-healing, stiff polymer, dynamic covalent bonds, siloxanes, recycling

## Abstract

As stiff polymers are difficult to self-heal, the balance between polymers’ self-healing ability and mechanical properties is always a big challenge. Herein, we have developed a novel healable stiff polymer based on the Si-OR and Si-OSi dynamic covalent bonds. The self-healing mechanism was tested and proved by the small molecule model experiments and the contrast experiments of polymers. This polymer possesses excellent tensile, bending properties as well as room temperature self-healing abilities. Moreover, due to the sticky and shapeable properties under wetting conditions, the polymer could be used as an adhesive. Besides, even after four cycles of recycling, the polymer maintains its original properties, which meets the requirements of recyclable materials. It was demonstrated that the polymer exhibits potential application in some fields, such as recyclable materials and healable adhesives.

## 1. Introduction

Self-healing behaviors in biology systems [1,2,3,4,5,6] have drawn scientists’ attention to develop various self-healing materials [7,8,9]. The first example of healable polymers was realized by Dry et al. [7,8]. A microcapsule-based self-healing polymer was created by Waite et al. [9] in 2001, which promoted the rapid development of research into polymer self-healing materials. Self-healing hydrogels [10,11,12] have been extensively explored. They often possess ultrafast self-healing [13,14,15,16], full-recovery [14,15,16] and rheological properties [17,18,19,20] without external stimuli. In contrast, the self-healing materials with strong mechanical properties usually require an external stimuli, such as a catalyst [21], heat [22], and pressure [23]. The sufficient chain mobility in polymers is the key factor to recover the original mechanical properties [24], which makes up the difference between stiff materials and soft hydrogels in self-healing ability. Thus, it is a big challenge to design and realize a polymer possessing both excellent mechanical and self-healing properties.

Recently, several works have realized the stiff and healable polymer systems via boroxine bonds [22,25], metal–ligand interactions [26,27], dynamic vinylogous urethane [28], dense hydrogen bonds [23] and Diels–Alder (DA) reactions [29]. Si-O exchange in siloxanes and silyl ethers have been employed to prepare dynamic cross-linked and self-healing Polydimethylsiloxane (PDMS) networks [30,31,32,33,34,35,36,37], rigid vitrimers [38,39,40,41], and recyclable thermoset plastics [42,43,44] as well as self-healing composites [45,46,47]. Therefore, it is important to take advantage of Si-OR and Si-OSi dynamic covalent bonds to explore healable polymers with a general strategy, high mechanical strength, mild healing conditions, recycling abilities and low cost.

**Figure 1:**
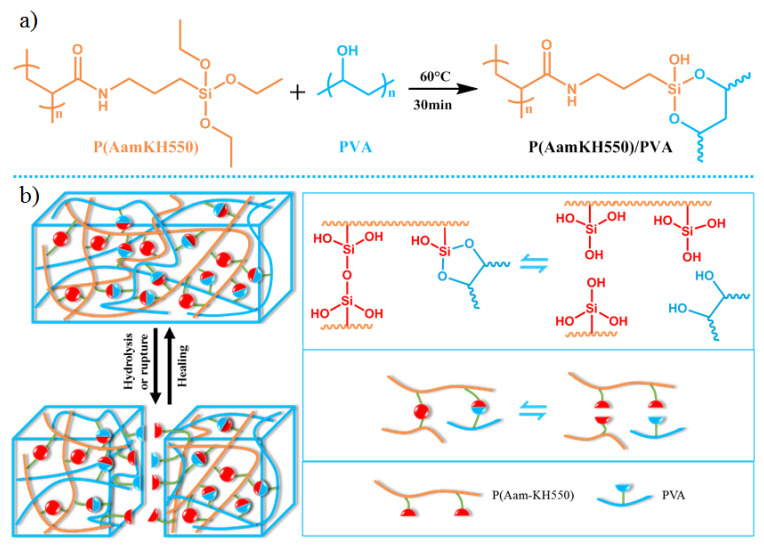
(**a**) The reaction route of **P(AamKH550)** (polymer of N-(4-(triethoxysilyl)butyl)acrylamide) and **PVA** (polyvinyl alcohol). (**b**) Proposed mechanism for self-healing of **P(AamKH550)/PVA** (polymer blends of P(AamKH550) and PVA).

Herein, we have reported a novel healable polymer based on the double exchange of silanol–alcohol and silanol–silanol dynamic covalent bonds. The cross-linked polymer P(AamKH550)/PVA consists of hard segments (siloxane part) and soft segments (polyvinyl part) [14,48]. This material exhibited high mechanical properties (with tensile strength up to 17.49 ± 0.56 MPa, tensile Young’s modulus up to 1187.68 ± 67.67 MPa and elongation up to 8.92 ± 0.19%, respectively) and self-healing properties (water-assisted self-healing, 72.8% at 25 °C and 99.6% at 70 °C recovery of strength). Apart from cross-linked chains in the polymer, Si-OR and Si-OSi dynamic covalent bonds could enhance the mechanical properties due to the contribution to polymer networks. When suffering rupture at room temperature, the Si-OR and Si-OSi dynamic covalent bonds shaped quickly with water assisting (Figure 1b). Over the glass-transition temperature (Tg), the Si-OR and Si-OSi dynamic covalent bonds and the sufficient chain mobility result in the full recovery of self-healing.

## 2. Materials and Methods

### 2.1. Materials

3-Aminopropyltriethoxysilane (KH550) and polyvinyl alcohol (PVA, degree of hydrolysis is 87.0–89.0 mol%, average Mn is 105600 and degree of polymerization is 2400) were purchased from Macklin. Polyacrylamide (PAam, average Mn is 10^6^) was purchased from Bidepharm. The remaining commercial reagents were used as received unless otherwise illustrated, and were purchased from Energy Chemical.

### 2.2. Structure Characterization

Solution ^1^H NMR (500MHz) and ^13^C NMR spectra (125 MHz) were recorded on a Bruker AVANCE Ⅲ 500 NMR spectrometer. Chemical shifts are reported in ppm relative to tetramethysilane as an internal standard (^1^H). The solvent was CDCl_3_. Solid-state ^13^C NMR spectra were recorded at 100 MHz on a Bruker AVANCE 400 spectrometer.

The molecular weight and polydispersity index (PDI) were recorded on a Waters Breeze 2 Gel Permeation Chromatograph. Flow rate of 1 mL/min, standard sample of polystyrene, mobile phase of tetrahydrofuran (THF) and 5 mg/mL THF solution of the test sample were adopted.

Fourier transform infrared spectra (FT-IR) were recorded on a NICOLET6700 fourier transform infrared spectrometer. The monomer was tested by a liquid pool method and the polymers were tested by a KBr pellet method.

Scanning electron microscopy image and SEM-EDS mapping were obtained on a scanning electron microscope (FEI/OXFORD/HKL Quanta 400) operated at an accelerating voltage of 3 kV.

X-ray photoelectron spectroscopy measurement was performed on a Nexsa XPS of Thermofisher Scientific with 12 kV, 720 W. The test sample was crushed and sieved through a 30 mesh screen.

### 2.3. Thermal Performance Tests

Thermogravimetric analysis curves (TG) were recorded on a TG209F1libra TG analyzer ranging from 35 °C to 800 °C under N_2_ with a heating rate of 10 °C/min. All samples were powder, which were crushed and sieved through a 30 mesh screen.

Differential scanning calorimetry curve (DSC) was recorded using a Netzsch DSC-204 DSC analyzer scanning from −40 °C to 150 °C under N_2_ with a 5 °C/min scanning rate. The sample was powder sieved through a 30 mesh screen.

### 2.4. Contact Angle Tests

Contact angle measurements were recorded on a KRUSS DSA25 Contact Angle meter under ambient conditions. The water absorbing measurements of P(AamKH550)/PVA (sample size: 40 mm length × 5 mm width × 2 mm height) were tested on a contact angle meter via a sessile drop method. The contact angles were recorded at 0 min, 3 min, 5 min, 7 min, 10 min, 14 min, 17 min, 25 min and 30 min.

### 2.5. Mechanical, Self-Healing and Recycling Tests

Mechanical tensile-stress and compressive-stress were tested on a single-column micro-control universal testing machine (Jingkong Mechanical Testing Co, Ltd, JK-500E, Guangzhou, China). For mechanical tensile-stress, self-healing and recycling measurements, a sample size of 40 mm length × 5 mm width × 2 mm height, gauge length of 20 mm, and strain rate of 10 mm/min were adopted. The humidity was controlled by a self-made chamber unless otherwise specified. For healing humidity measurements, samples were put into chambers at room temperature with different relative humidity (23%, 43% and 75%). For healing time measurements, samples self-repaired under the same conditions (23% relative humidity and 25 °C) for different healing time (1 h, 3 h, 6 h, 12 h and 24 h). For healing temperature measurements, self-healing samples were put into a temperature humidity chamber at an optimum relative humidity at 25 °C, 50 °C, 60 °C and 70 °C respectively. For recycling measurements, fractured samples after tensile-stress tests were reprocessed at 60 °C and 15 MPa for 20 min, which were repeated at least four times. Moreover, for bend measurements, a sample size of 50 mm length × 5 mm width × 2 mm height, gauge length of 20 mm, and a strain rate of 2 mm/min were adopted. Every measurement was tested for at least three parallel samples at ambient conditions and the average was recorded.

A digital metallographic microscope (Weiscope WSM500D, Guangzhou, China) was used to record the self-healing process of the sample (sample size: 40 mm length × 5 mm width × 2 mm height).

The photographs and movie of samples were taken by camera (Canon EOS, Beijing, China). The sample sizes were listed in each figure caption.

### 2.6. Adhesion Tests

The adhesive properties of the samples were tested on a single-column micro-control universal testing machine (Jingkong Mechanical Testing Co, Ltd., JK-500E, Guangzhou, China). Strain rate 5 mm/min and gauge length 125 mm were adopted. Every measurement was tested in at least three parallel samples at ambient conditions and the average was recorded. The sample was applied evenly to the surface of stainless steel sheets after sandpaper grinding. The curing area was connected and dried for 24 h. The size of the curing area was 12.5 mm length × 25 mm width. The curing temperature measurement was tested after sample healing at different temperatures for 24 h.

### 2.7. Mechanism Studies

#### 2.7.1. In-Situ IR

The reaction processes at different reaction times of siloxane and alcohol were noted on an In-Situ IR (METTLER TOLEDO ReactIR ic10, America). 3-aminopropyltriethoxysilane (KH550) (2.0000 g) and ethylene glycol (2.0000 g) were added into water (5 mL) and then stirred at room temperature. The trend of peak intensity and peak position of the target functional group over time was monitored by In-Situ IR.

#### 2.7.2. ESI-MS

The molecular weights of small molecules were recorded on a mass spectrometer (Thermofisher TSQ Quantum Ultra, ESI source, America). 3-aminopropyltrimethoxysilane (KH540) (0.8964 g, 5 mmol) and 3-aminopropyltriethoxysilane (KH550) (1.1068 g, 5 mmol) were mixed for 30 min at 25 °C.

#### 2.7.3. ^1^H NMR

The KH550 was mixed with CD_3_CD_2_OD at a mass ratio of 1:1. After 10 minutes of reaction, the change in the integral area of characteristic peaks in the reaction system was tested by a Bruker AVANCE 400 NMR spectrometer. The test temperature was 25 °C and the scanning range was between −3 ppm and 16 ppm.

### 2.8. Preparation of P(AamKH550)/PVA

P(AamKH550) was prepared by radical polymerization of monomer N-(4-(triethoxysilyl)butyl)acrylamide (AamKH550). **P(AamKH550)/PVA** was obtained by reaction of P(AamKH550) and PVA. The aqueous solution of 20 wt.% PVA (5.1889 g) was obtained by stirring in a 100 mL round bottom flask at 90 °C for 3 h. The tetrahydrofuran solution of P(AamKH550) (5.1889 g) was added dropwise into the aqueous solution of PVA at 60 °C stirring for 30 min. The pale-yellow gelatinous substance was collected and dried overnight under vacuum. ^13^C SSNMR (100 MHz): δ = 177.64 (s), 58.27 (s), 42.04 (s), 34.37 (s), 23.42 (s), 18.42 (s), 9.75 (s) ppm (Supplementary Materials, Figure S3a-A).

## 3. Results and Discussions

### 3.1. Structural Characterization of P(AamKH550)/PVA

**Figure 2:**
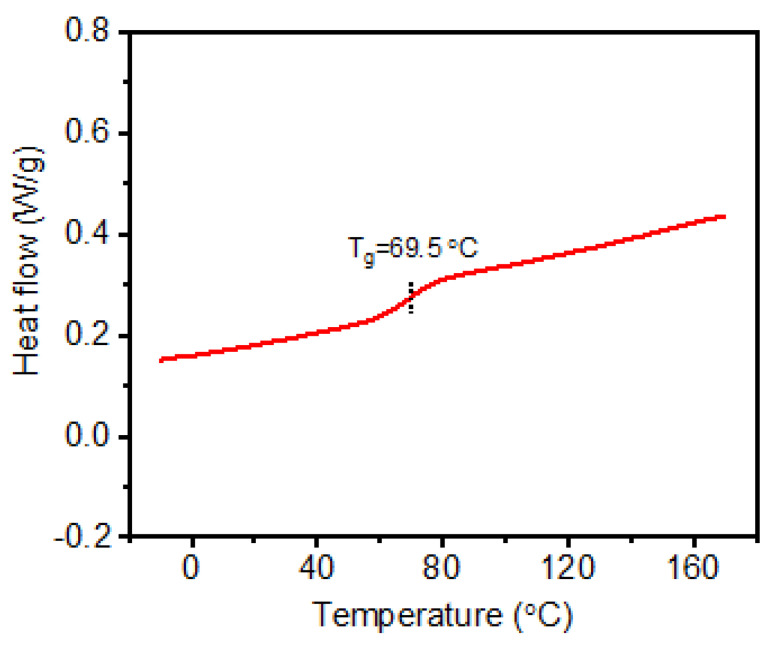
Differential scanning calorimetry (DSC) curve of **P(AamKH550)/PVA**.

P(AamKH550) was the polyacrylamide terminated with silane coupling agent, which could hydrolyze to silanol and then form bonds with alcohol hydroxyl in PVA (-Si-OR). The –Si-OR bonds formed cross-linked networks in **P(AamKH550)/PVA**. At the same time, the –Si-OSi bonds were formed by dehydrolysis of –Si-OH. Solid-state ^13^C NMR (Figure S3a) and FT-IR (Figure S3b) were used to demonstrate the successful preparation **P(AamKH550)/PVA**. SEM image and EDS phase mapping (Figure S4) showed that the C, Si, and O elements were evenly distributed on the surface of **P(AamKH550)/PVA**. XPS tests (Figure S5) demonstrated that the Si element forms covalent bonds in **P(AamKH550)/PVA**. Thermogravimetric analysis (TGA) measurements indicated that **P(AamKH550)/PVA** is more thermally stable than P(AamKH550) (Figure S6). The glass-transition temperature (Tg) of **P(AamKH550)/PVA** is 69.5 °C through differential scanning calorimetry (DSC) measurements (Figure 2), which demonstrated P(AamKH550)/PVA is homogeneous. Furthermore, to reveal that the Si-O equilibrium plays a constructive role in the self-repairing process, **P(AamKH550-Aam)/PVA** (copolymers of AamKH550 and Aam cross-linking with PVA) and **PAam/PVA** [49,50] (polymer blends of PAam and PVA) were prepared at the same conditions and then characterized by FT-IR (Figure S3b).

### 3.2. Mechanical Properties of P(AamKH550)/PVA

**Figure 3:**
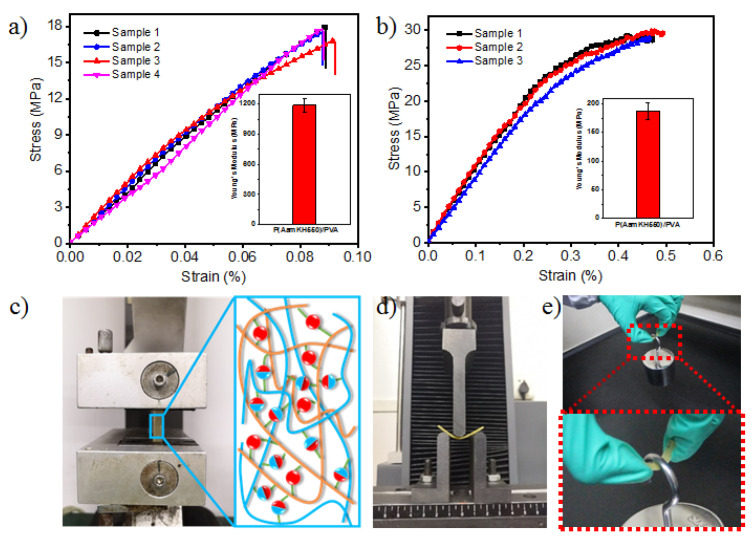
Mechanical properties of **P(AamKH550)/PVA** strips. (**a**) Mechanical tensile-stress curves of **P(AamKH550)/PVA** samples tested under ambient conditions (25 °C, 45H%). The illustration shows the average and the error bar of tensile Young’s modulus of the tested four samples. (**b**) Mechanical bending-stress curves of **P(AamKH550)/PVA** samples tested under ambient conditions (25 °C, 45H%). The illustration shows the average and error bar of bending Young’s modulus of the measured three samples. (**c**) The image of tensile-stress tests of **P(AamKH550)/PVA**. (**d**) The image of bending-stress tests of **P(AamKH550)/PVA**. (**e**) The image of 0.2612 g strip of **P(AamKH550)/PVA** withstanding a 1 kg hook weight (sample size: 40 mm length × 5 mm width × 2 mm height).

The mechanical properties of **P(AamKH550)/PVA** were measured via a static uniaxial tensile test [22,25]. The **P(AamKH550)/PVA** strips demonstrate an incredibly high tensile Young’s modulus up to 1187.68 ± 67.67 MPa and an excellent tensile strength up to 17.49 ± 0.56 MPa (Figure 3a, Table S1). Besides, the elongation of the strips (8.92 ± 0.19%) exhibits the stiff and strong feature of the material. To further prove the stiff property of the polymer, the bend measurements were carried out under an ambient air, resulting in a high bending Young’s modulus and a bending strength up to 187.39 ± 15.27 MPa and 29.67 ± 0.49 MPa, respectively (Figure 3b, Table S2). Further exploration found that the sample could withstand 1 kg, about 5000 times their own weight (Figure 3e). The high Young’s modulus and excellent mechanical properties should be ascribed to the high degree of the cross-linked networks within the novel material.

### 3.3. Self-healing Properties of P(AamKH550)/PVA

**Figure 4:**
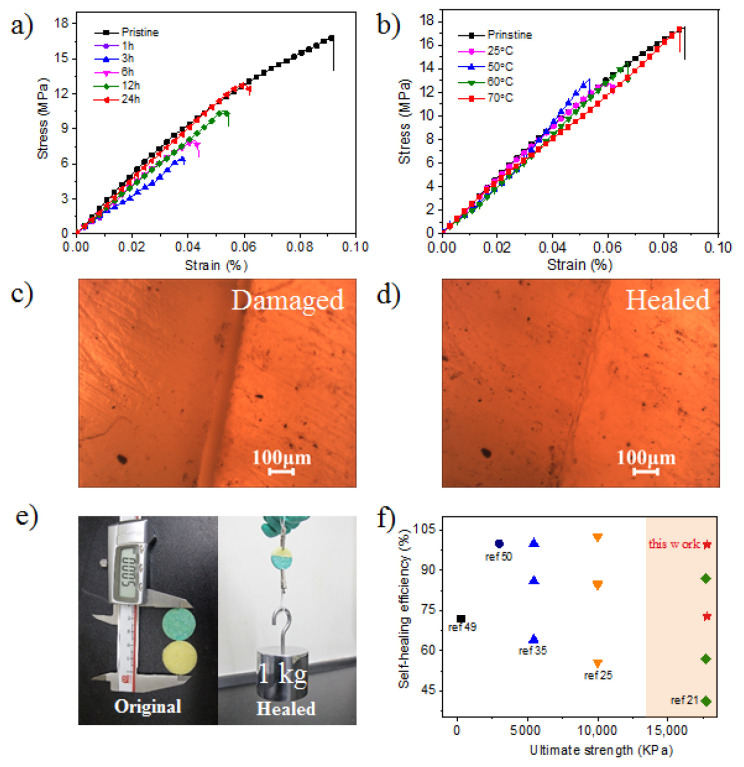
Self-healing properties of **P(AamKH550)/PVA** strips. (**a**) Mechanical tensile-stress curves of the pristine **P(AamKH550)/PVA** samples and healed samples at 25 °C and 23H% for 1 h, 3 h, 6 h, 12 h and 24 h, respectively. (**b**) Mechanical tensile-stress curves of the pristine **P(AamKH550)/PVA** samples and **P(AamKH550)/PVA** samples healed for 24 h at 23H% and 25 °C, 50 °C, 60 °C and 70 °C, respectively. (**c**) Optical microscopic image of a damaged **P(AamKH550)/PVA** strip under ambient conditions. (**d**) Optical microscopic image of the **P(AamKH550)/PVA** strip healed for 1 h under ambient conditions. (**e**) Photographs of original **P(AamKH550)/PVA** disks in 25 mm diameter (left) and healed **P(AamKH550)/PVA** disks (right) after healing (25 °C, 23H%) for 1 h withstanding 1 kg of weight. (**f**) Comparison of the healable materials in this work and recent reports on the self-healing efficiency and ultimate strength.

Self-healing properties of the material were further measured by the recovery of mechanical properties. It is obvious that healing humidity (percent of humidity during healing), healing time and healing temperature have a massive effect on the healing efficiency. The self-healing process contained several steps: The strip samples were initially cut into two pieces with a paper knife, then two sections were wetted by deionized water momently and were put together. The healing samples were settled in different containers to explore the effects of healing humidity, healing time and healing temperature in sequence. After a quantitative studies of these factors, prolonging healing time (Figure 4a, Table S5) or raising the healing temperature (Figure 4b, Table S4) could increase the healing efficiency. However, the healing efficiency would increase by decreasing the healing humidity (Figure S7, Table S3). A healing efficiency at 23H% (H% means percent of relative humidity) for 24 h would reach 73% with a tensile strength of up to 12.74 MPa (Figure 4a) (tensile Young’s modulus about 1258.05 MPa). When the healing temperature is raised to 70 °C for 24 h, the mechanical properties of the polymer sample would completely recover with the tensile strength, up to 17.42 MPa (Figure 4b) and tensile Young’s modulus up to 1110.74 MPa, 93% of the original samples. This means that the healing efficiency could reach up to 99% at 70 °C. When the two cracked sections contacted each other, they would quickly establish a connection again. As shown in the Figure 4c,d, the notch on cracked samples almost disappeared after self-healing (25 °C, 23H%) for 1 h, which could be observed under the optical microscope. Then two yellow polymer disks were prepared and one of the polymer disks was colored in blue with methylene blue trihydrate (Figure 4e). After being cut into two sections, one of each of the polymer disk sections were put together. The healed sample could withstand 1 kg as 1000 times its own weight easily after healing for 1 h at room temperature. The **P(AamKH550)/PVA** were proved to have good self-healing abilities, even under mild conditions. A demonstration video for the self-healing process of a strip was presented in the Movie S1. Furthermore, the healing efficiency and the ultimate strength of the material we developed and previous reports were compared and the result is shown in Figure 4f, among which **P(AamKH550)/PVA** shows a good performance.

### 3.4. Self-healing Mechanisms of P(AamKH550)/PVA

**Figure 5:**
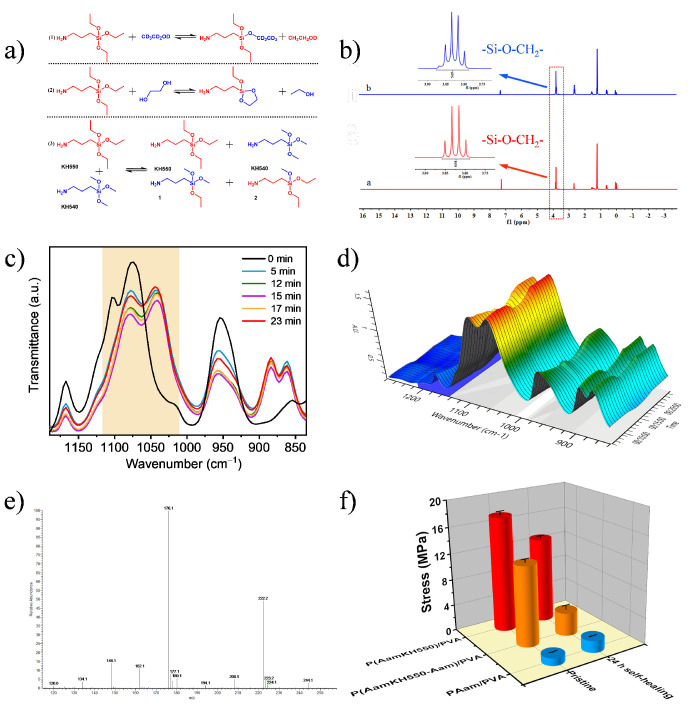
Self-healing mechanism studies of **P(AamKH550)/PVA**. (**a**) Schemes of ^1^H NMR (1), In-Situ IR (2), ESI-MS (3). (**b**) ^1^H NMR spectra of 3-aminopropyltriethoxysilane in CDCl_3_ (spectrum a) and the exchange reaction of 3-aminopropyltriethoxysilane with ethanol-D_6_ in CDCl_3_ (spectrum b). The amplified insets show integral area of –Si-O-CH_2_- of spectrum a and b, respectively. (**c**) In-Situ IR spectra of the reaction between aminopropyltriethoxysilane (KH550) and ethylene glycol. (**d**) The three-dimensional in-situ IR spectra of the reaction between KH550 and ethylene glycol. (**e**) ESI-MS spectra of exchange reaction between 3-aminopropyltrimethoxysilane (KH540) and KH550 at 25 °C. (**f**) Healing properties of **P(AamKH550)/PVA**, **P(AamKH550-Aam)/PVA**, **PAam/PVA** strips healed at 25 °C and 23H% for 24 h.

To deeply understand and prove the self-healing mechanism of **P(AamKH550)/PVA**, we took advantage of small-molecule model experiments together with the macromolecules’ contrast experiments. The small-molecule model experiments (Figure 5a) were designed to study the two equilibriums of silanol-alcohol and silanol-silanol, as well as the role of water in the self-healing process via ^1^H NMR [38], In-Situ IR, and ESI-MS measurements. When exchanging two types of silane coupling agents at 25 °C, 3-aminopropyltrimethoxysilane (KH540) and 3-aminopropyltriethoxysilane (KH550), brands of new molecular weights, were tested by the ESI-MS measurement. The ESI-MS spectra (Figure 5a(3),e and Figure S8b) demonstrated that KH550 and KH540 would exchange without any external force and then new types of silane coupling agents were formed. Through the In-Situ IR spectra, it was also found that the reaction between alcohol and the silane coupling agent generated an evident wavenumber movement and a regular change in the peak intensity of –Si-O and –Si-C (Figure 5a(2),c,d). Comparing the original tensile strength and self-healing properties of homopolymer **P(AamKH550)/PVA**, copolymer P(AamKH550-Aam)/PVA and mixture **PAam/PVA** (Figure 5f and Table S6) further brought support to the self-healing mechanism that the silanol-alcohol and silanol-silanol exchanging interactions did indeed play a main role in the self-healing properties within the material **P(AamKH550)/PVA**. When suffering rupture, the Si-OR and Si-OSi dynamic covalent bonds shaped quickly with water assisting, and the Si-OR and Si-OSi dynamic covalent bonds would then shift towards silyl ethers by water releasing, and finally, the polymer networks were reformed with a recovery of the mechanical properties. When cracking at room temperature, the Si-OR and Si-OSi dynamic covalent bonds formed quickly with water assistance. To be specific, Si-OH and R-OH would form via water wetting and then Si-OH would form bonds with Si-OH and R-OH through dehydration. Finally, the polymer networks self-heal with the recovery of mechanical properties. Over the Tg, the Si-OR and Si-OSi dynamic covalent bonds and the sufficient chain mobility results in the full recovery of self-healing.

### 3.5. Recycling Properties of P(AamKH550)/PVA

**Figure 6:**
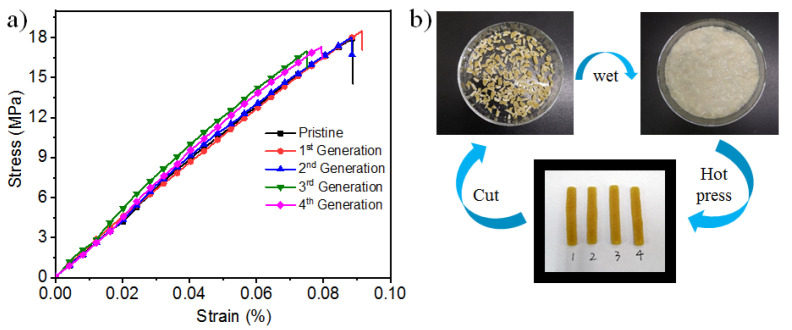
Recycling properties of **P(AamKH550)/PVA**. (**a**) Tensile-stress curves of the pristine **P(AamKH550)/PVA** strips and reprocessing **P(AamKH550)/PVA** strips after the 1st, 2nd, 3rd and 4th generation. (**b**) Photographs of the reprocessing method of the **P(AamKH550)/PVA** strips.

Despite these polymers being stiff and strong, to our delight, the material **P(AamKH550)/PVA** could be reprocessed and recycled (Figure 6b). When the original samples suffered fracture during the tensile measurements, they were cut into small pieces and wetted by water to a uniform viscosity. Then, the sticky samples were hot-pressed into rectangle samples under a certain humidity. Although the polymer networks were stable over the glass-transition temperature (Tg), they would be swollen and would then absorb water quickly, which were proven by the water contact angle measurements (Figure S9a,b) and the FT-IR spectra of the dry and wet samples (Figure S9c). The self-healing mechanism could explain the phenomenon that water would promote the –Si-OSi and –Si-OR equilibrium moving to –Si-OH and –R-OH, which would enable the polymer 3D networks to reconstruct. Thus, the hot-press would form a new generation of **P(AamKH550)/PVA,** maintaining its mechanical property with a slight decrease after four cycles of reprocessing, as shown in Figure 6a and Table S7, which demonstrates that **P(AamKH550)/PVA** possesses good stable and recycling properties and could be used as a potential recyclable material.

### 3.6. Adhesive Property of P(AamKH550)/PVA

**Figure 7:**
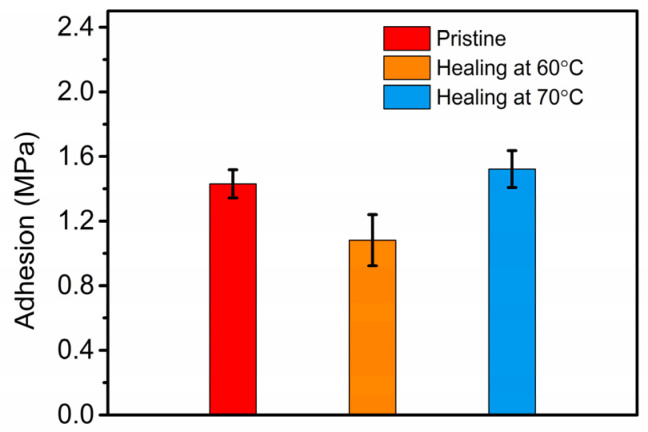
Adhesion tests of P(AamKH550)/PVA.

Moreover, it was found that **P(AamKH550)/PVA** was turned to be sticky after wetting by water. Therefore, adhesion test samples of the **P(AamKH550)/PVA** were prepared with water-wetting and were tested after having dried fully. The average adhesion strength of the pristine sample results in 1.43 ± 0.08 MPa (Figure 7). The adhesion strengths healed at 60 °C and 70 °C for 24 h, respectively, and the exhibited curing effects differed from healing temperature [25]. At 60 °C, the healed strength could repair up to 76% of the original sample. It could completely recover at 70 °C. Combined with the self-healing mechanisms of **P(AamKH550)/PVA** in Section 3.4, we know that **P(AamKH550)/PVA** has a good chain mobility when the healing temperature reached up to Tg (69.5 °C); at the same time, -Si-OR and –Si-OSi could establish a covalent bond connection again. The dual functions promote the healing process of the rupture surface. The adhesion tests demonstrate that **P(AamKH550)/PVA** could be used as a healable adhesive.

## 4. Conclusions

In conclusion, we have developed a room-temperature self-healing polymer **P(AamKH550)/PVA**, with high mechanical properties by water-assistance. Si-OR and Si-OSi dynamic covalent bonds endue the polymer networks with excellent tensile, bending, self-healing and recycling properties. Thus, this stiff polymer could be reprocessed and recycled under mild conditions owing to the water-adsorption capacity, as well as rebuilding three-dimensional cross-linking networks. Moreover, **P(AamKH550)/PVA** turns sticky and malleable after water absorbance, which reveals potential in the field of recyclable materials and healable adhesives. Further exploration in this area is being performed in our lab.

## Data Availability

The data presented in this study are available on request from the corresponding author.

## Supplementary Materials

The following are available online at https://www.mdpi.com/article/10.3390/ma14102680/s1, Figure S1. Synthesis schemes for AamKH550 and NMR spectra of AamKH550. (a) 1H NMR spectrum of AamKH550. (b) 13C NMR spectrum of AamKH550; Figure S2. (a) Synthesis schemes for P(AamKH550), P(AamKH550-Aam). (b) Reaction photographs of P(AamKH550) in THF and PVA in deionized water. (c) FT-IR spectrum of AamKH550, P(AamKH550), P(AamKH550-Aam).; Figure S3. Synthesis schemes for P(AamKH550)/PVA, P(AamKH550-Aam)/PVA, PAam/PVA, and NMR and FT-IR spectra of P(AamKH550)/PVA, P(AamKH550-Aam)/PVA, PAam/PVA. (a) 13C SSNMR spectrum of P(AamKH550) and P(AamKH550)/PVA. (b) FT-IR spectrum of P(AamKH550)/PVA, P(AamKH550-Aam)/PVA and PAam/PVA; Figure S4. (a) SEM images and EDS phase mapping of the distribution of elements in fracture section of P(AamKH550)/PVA (C: blue, O: green, Si: red). (b) SEM image of P(AamKH550)/PVA; Figure S5. XPS pattern of P(AamKH550)/PVA; Figure S6. Thermogravimetric analysis (TG) curves of P(AamKH550) and P(AamKH550)/PVA; Figure S7. Healing humidity studies of P(AamKH550)/PVA; Figure S8. (a) ^1^H NMR spectra of 3-aminopropyltriethoxysilane in CDCl_3_ (spectrum a) and the exchange reaction of 3-aminopropyltriethoxysilane with ethanol-D_6_ in CDCl_3_ (spectrum b). The amplified insets show the integral area of –CH3 of spectrum a and b, respectively. (b) ESI-MS spectra of exchange reaction between 3-aminopropyltrimethoxysilane (KH540) and 3-aminopropyltriethoxysilane (KH550) at 25 °C; Figure S9. Water absorbing ability of P(AamKH550)/PVA. (a) The contact angle measurements of P(AamKH550)/PVA strip. (b) Images of contact angle measurements at 0 min, 3 min, 5 min, 7 min, 10 min, 14 min, 17 min, 25 min, 30 min. (c) IR spectra of dry and wet P(AamKH550)/PVA; Figure S10. GPC trace of P(AamKH550); Table S1. Data of mechanical tensile property of P(AamKH550)/PVA; Table S2. Data of mechanical bending property of P(AamKH550)/PVA; Table S3. The effect of healing humidity of P(AamKH550)/PVA; Table S4. The effect of healing temperature of P(AamKH550)/PVA; Table S5. The effect of healing time of P(AamKH550)/PVA; Table S6. The comparison data of mechanical properties and healing properties of P(AamKH550)/PVA, P(AamKH550-Aam)/PVA, PAam/PVA; Table S7. The recycling properties of P(AamKH550)/PVA; Table S8. The molecular weight and PDI of P(AamKH550).
