# Supplementary material for: Dynamic Covalent Bonds of Si-OR and Si-OSi Enabled A Stiff Polymer to Heal and Recycle at Room Temperature
Source: Materials (Basel). 2021 May 20;14(10):2680. doi: 10.3390/ma14102680 (PMC8160654; doi:10.3390/ma14102680)
Supplement: Supplementary file 1 [file materials-14-02680-s001.zip › materials-1197736-Supplementary.pdf]

Supporting Information

Dynamic Covalent Bonds of Si-OR and Si-OSi Enabled A Stiff Polymer to Heal and Recycle at Room Temperature

Ping Fan,[1] Can Xue,*[2] Xiantai Zhou,[2] Zujin Yang,[2] Hongbing Ji*[1,2,3]

Affiliations:

(1) Fine Chemical Industry Research Institute, School of Chemistry, Sun Yat-Sen University, Guangzhou, 510275, P. R. China, E-mail: jihb@mail.sysu.edu.cn ORCID 0000-0003-1684-9925

(2) Fine Chemical Industry Research Institute, School of Chemical Engineering and Technology, Sun Yat-Sen University, Zhuhai 519082, P. R. China, E-mail: xuecan@mail.sysu.edu.cn ORCID 0000-0002-2425-7922

(3) Maoming Branch, Guangdong Laboratory for Lingnan Modern Agriculture, Maoming 525000, P. R. China

Table of Contents

Table of Contents 2
1. Material and methods 2
1.1 Synthesis of AamKH550 2
1.2 Synthesis of P(AamKH550), P(AamKH550-Aam) 3
1.3 Synthesis of P(AamKH550-Aam)/PVA, PAam/PVA 5
2. Supplementary Figures 6
3. Supplementary Tables 10
References 13

1. Material and methods

1.1 Synthesis of AamKH550

AamKH550 was prepared according Florentiev[1] reporting. Acryloyl chloride (9.5025 g, 105 mmol) and 250 mL anhydrous dichloromethane were added to a 500 mL round bottomed flask in an ice bath. 3-aminopropyltriethoxysilane (22.1370 g, 100 mmol) and anhydrous triethylamine was dissolved in 50 mL anhydrous dichloromethane and was then added dropwise into a stirred acryloyl chloride solution at 0°C under dry nitrogen for 6 h. The reacted mixture was filtered to remove the generated salt and then the filtrate was distillated to remove dichloromethane. Next, the solid–liquid mixture was extracted by hexane (150 mL), and a yellow sticky liquid was collected by filtration and distillation (yield 72%). ^{1}H NMR (500 MHz; $CDCl_3$): δ = 6.2 (d, J = 1.5 Hz, 1H; -NH-), 6.2 (d, J = 1.5 Hz, 1H; -CH=), 6.0 (dd, J_1 = 17.0 Hz, J_2 = 10.2 Hz, 1H; =CH_2), 5.5 (dd, J_1 = 10.2, J_2 = 1.5 Hz, 1H; =CH_2), 3.8 (q, J = 7.0 Hz, 6H; -O-CH_2-), 3.3 (q, J = 6.8 Hz, 2H; -NH-CH_2-), 1.56-1.62 (m, 2H; -CH_2-), 1.2 (t, J = 7.0 Hz, 9H; -CH_3), 0.61–0.55 (m, 2H; -Si-CH_2-) ppm; (Figure S1a). ^{13}C NMR (125 MHz; $CDCl_3$): δ = 165.55, 131.14, 125.85, 58.44, 41.84, 22.78 ppm (Figure S1b).

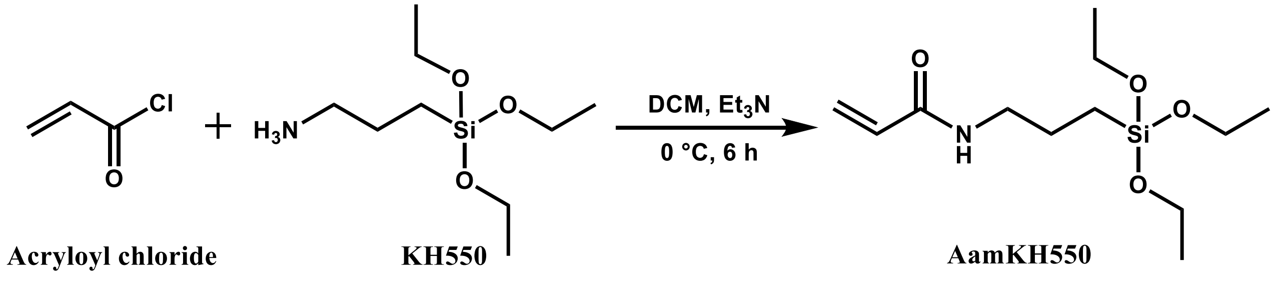

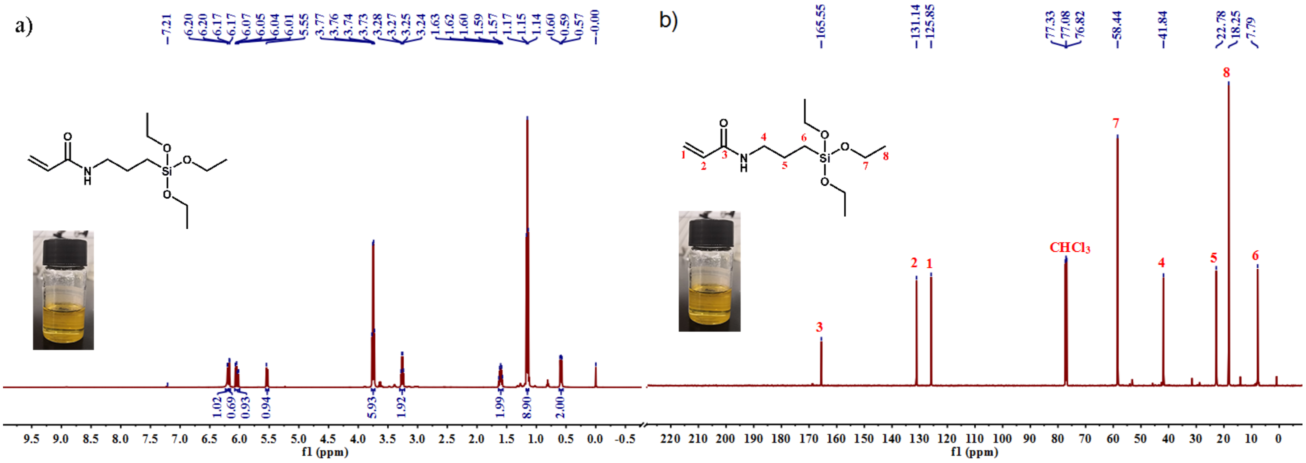

Figure S1 Synthesis schemes for AamKH550 and NMR spectra of AamKH550. a) ^{1}H NMR spectrum of AamKH550. b) ^{13}C NMR spectrum of AamKH550.

1.2 Synthesis of P(AamKH550), P(AamKH550-Aam)

P(AamKH550) was prepared by a radical polymerization[2] initiated by 2,2'-azobis(2-methylpropionitrile) (AIBN), in particular, AIBN was purified by recrystallization with ethanol. AamKH550 (8.2626 g, 30 mmol) and AIBN (0.0827 g, 1 wt.% of AamKH550) were added into a 500 mL round bottom flask successively and 300 mL anhydrous tetrahydrofuran (300 ml) as a solvent was added to the flask. The mixture was stirred for 3 h at 70°C under nitrogen, and then the temperature was increased to 80°C. After 4 h reacting at 80°C, another 1 wt.% AIBN (0.0827 g) was added into the flask. The reaction lasted a total of 24 h, quenched by cooling down to room temperature. The mixture was concentrated by distillation and then a pale-yellow solid (yield 63%) was obtained by precipitation with hexane (200 mL) and was dried for 24 h under vacuum. ^{13}C SSNMR (100 MHz): δ = 175.37 (s), 57.40 (s), 41.54 (s), 22.27 (s), 17.56 (s), 8.80 (s) ppm (Figure S3a-b). **P(AamKH550-Aam)** was prepared in the same conditions as **P(AamKH550)** with 1 equivalent Aam and 1 equivalent AamKH550 (yield 80%). AamKH550, **P(AamKH550)**, and **P(AamKH550-Aam)** were measured by FT-IR. FT-IR (KBr): ν = 3435, 2937, 1660, 1545, 1068, 906 cm^{-1} (Figure S2c).

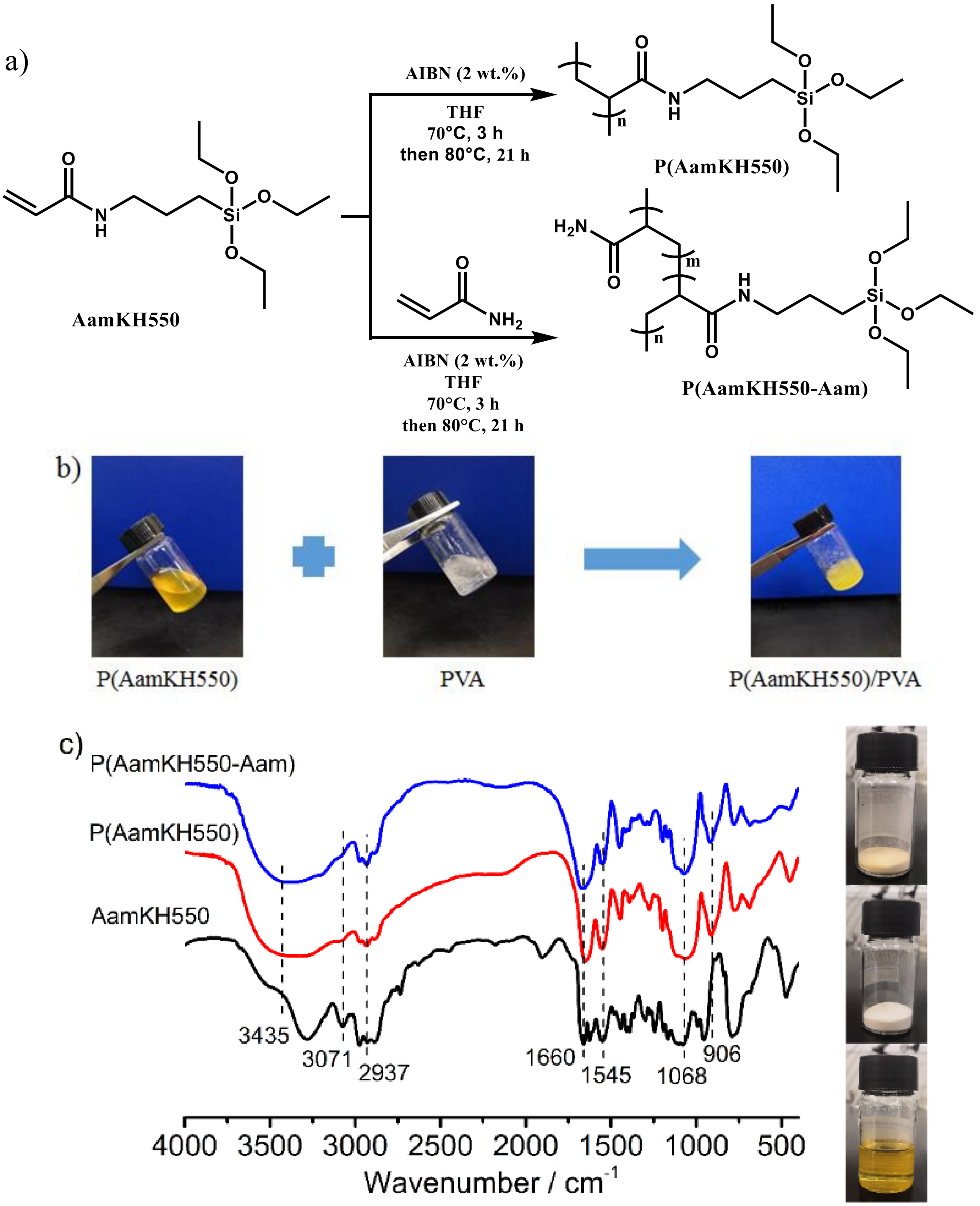

Figure S2 a) Synthesis schemes for **P(AamKH550)**, **P(AamKH550-Aam)**. b) Reaction photographs of **P(AamKH550)** in THF and PVA in deionized water. c) FT-IR spectrum of **AamKH550**, **P(AamKH550)**, **P(AamKH550-Aam)**.

1.3 Synthesis of P(AamKH550-Aam)/PVA, PAam/PVA

P(AamKH550-Aam)/PVA was obtained by reaction of **P(AamKH550-Aam)** and PVA. The aqueous solution of 20 wt.% PVA (5.1889 g) was obtained by stirring in a 100 mL round bottom flask at 90°C for 3 h. The tetrahydrofuran solution of **P(AamKH550-Aam)** (5.1889 g) was added dropwise into the aqueous solution of PVA at 60°C stirred for 30 min. The solid was collected and dried overnight under vacuum. *PAam/PVA*[3] was obtained by mixing of **PAam** (5.1889 g) and PVA (5.1889 g) via mechanical stirring for 24 h at room temperature. Comparison of **P(AamKH550)/PVA**, **P(AamKH550-Aam)/PVA**, and **PAam/PVA** was observed by FT-IR. FT-IR (KBr): ν = 1051, 916 cm^{-1} (Figure S3b).

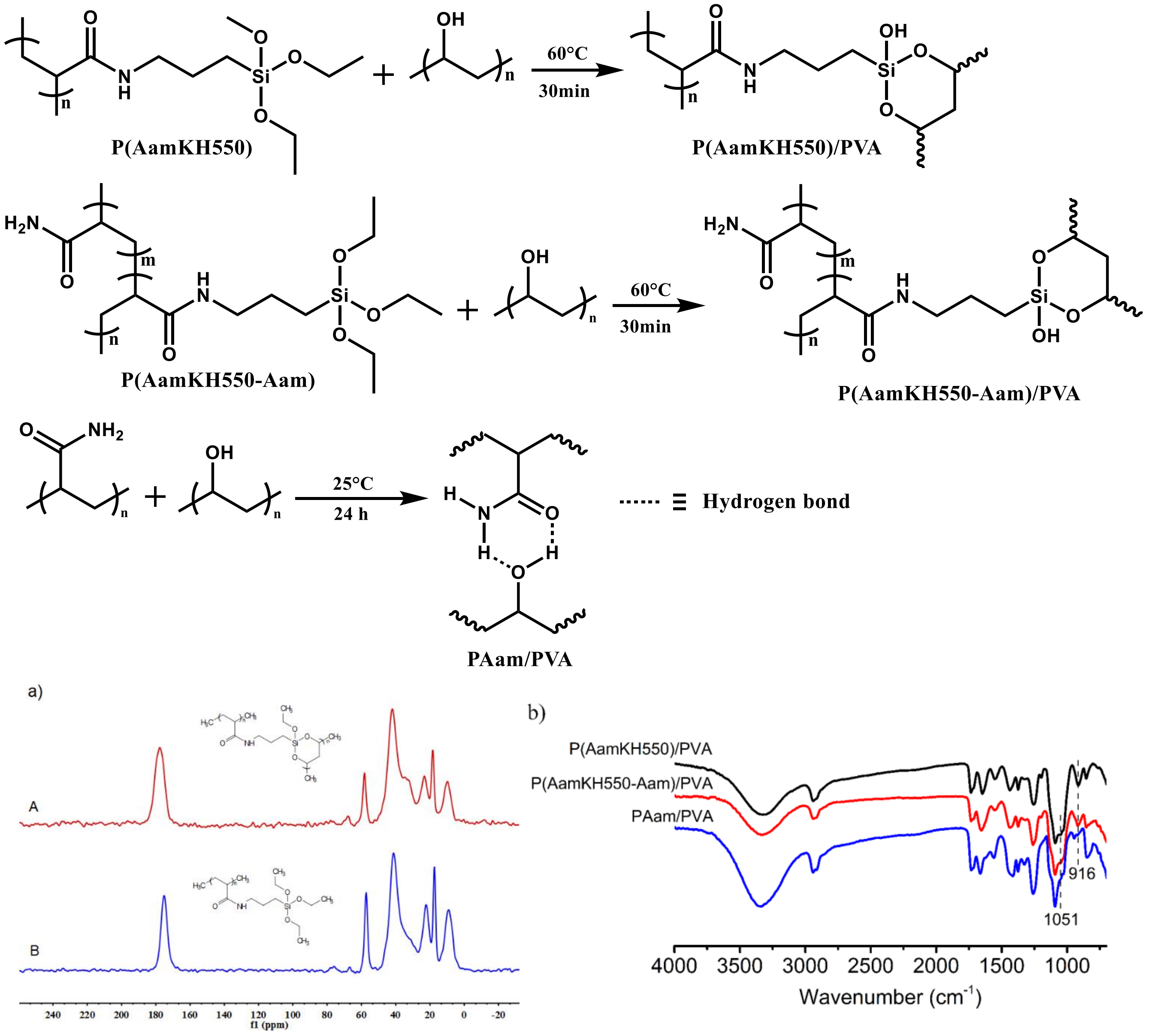

Figure S3 Synthesis schemes for **P(AamKH550)/PVA**, **P(AamKH550-Aam)/PVA**, **PAam/PVA**, and NMR and FT-IR spectra of **P(AamKH550)/PVA**, **P(AamKH550-Aam)/PVA**, **PAam/PVA**. a) ^{13}C

SSNMR spectrum of **P(AamKH550)** and **P(AamKH550)/PVA**. b)FT-IR spectrum of **P(AamKH550)/PVA**, **P(AamKH550-Aam)/PVA** and **PAam/PVA**.

2. Supplementary Figures

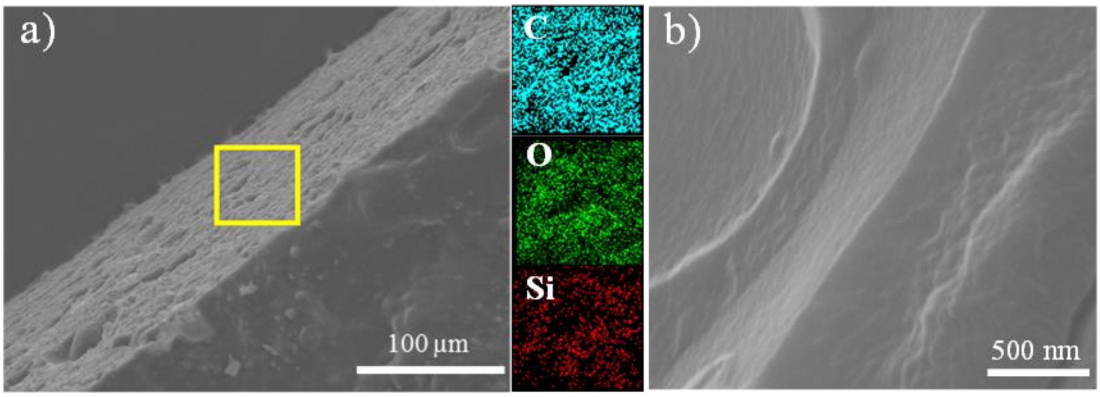

Figure S4 a) SEM images and EDS phase mapping of the distribution of elements in fracture section of P(AamKH550)/PVA (C: blue, O: green, Si: red). b) SEM image of P(AamKH550)/PVA.

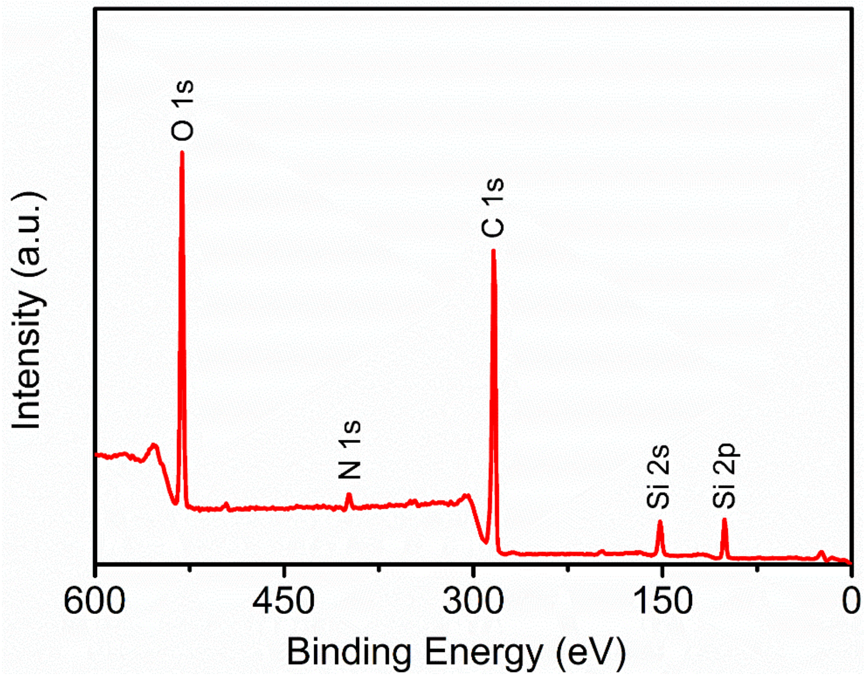

Figure S5 XPS pattern of P(AamKH550)/PVA.

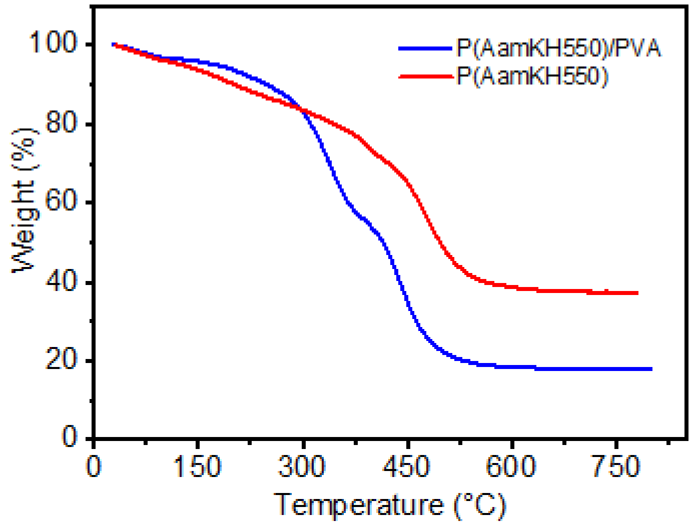

Figure S6 Thermogravimetric analysis (TG) curves of **P(AamKH550)** and **P(AamKH550)/PVA**.

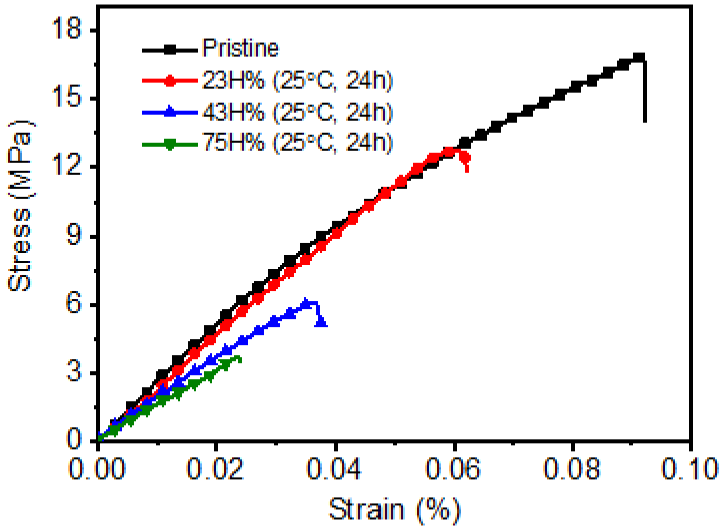

Figure S7 Healing humidity studies of **P(AamKH550)/PVA**.

Mechanism studies in ESI-MS measurement

3-aminopropyltrimethoxysilane (KH540) (0.8964 g, 5 mmol) and 3-aminopropyltriethoxysilane (KH550) (1.1068 g, 5 mmol) were mixed for 30 min at 25°C (Figure 4e). The exchanging products of siloxane bonds were obtained via ESI-MS spectra (Figure 4e) with molecular weights 180.1, 194.1, 208.1 and 222.2 belonging to KH540, molecule 1, molecule 2, and KH550 respectively. As shown in Figure S6b, many high molecular weight products were observed, which further proved the exchanging reaction between siloxane bonds.

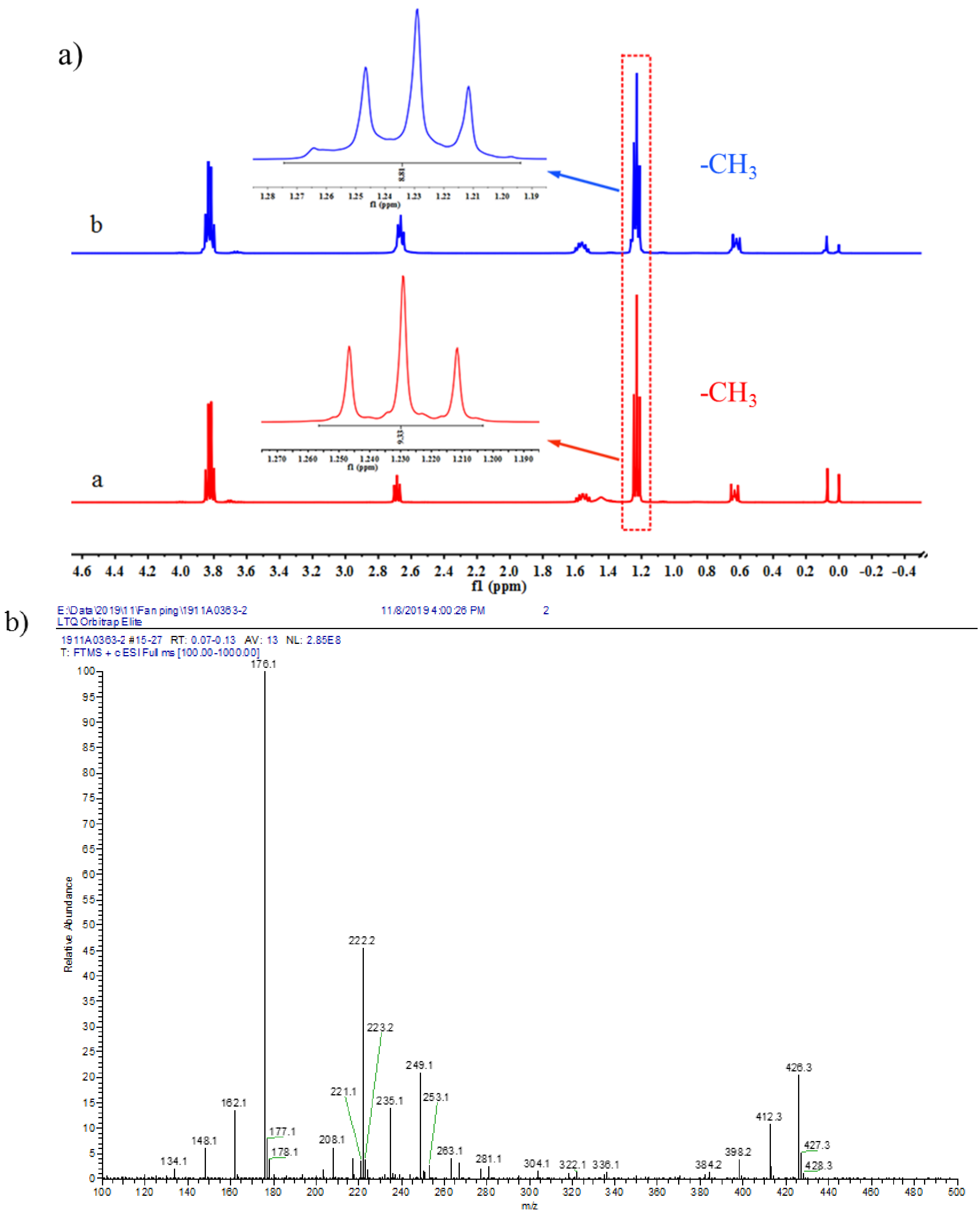

Figure S8 a) ^{1}H NMR spectra of 3-aminopropyltriethoxysilane in $CDCl_3$ (spectrum a) and the exchange reaction of 3-aminopropyltriethoxysilane with ethanol-D_6 in $CDCl_3$ (spectrum b). The amplified insets show the integral area of –CH_3 of spectrum a and b, respectively. b) ESI-MS spectra of exchange reaction between 3-aminopropyltrimethoxysilane (KH540) and 3-aminopropyltriethoxysilane (KH550) at 25°C.

Water absorbing ability measurements

As shown in Figure S9a and S9b, the decreasing contact angle indicated the good water absorbing ability of **P(AamKH550)/PVA**. Besides, IR spectra (Figure S9c) showing the difference between dry and wet **P(AamKH550)/PVA** further proved the conclusion.

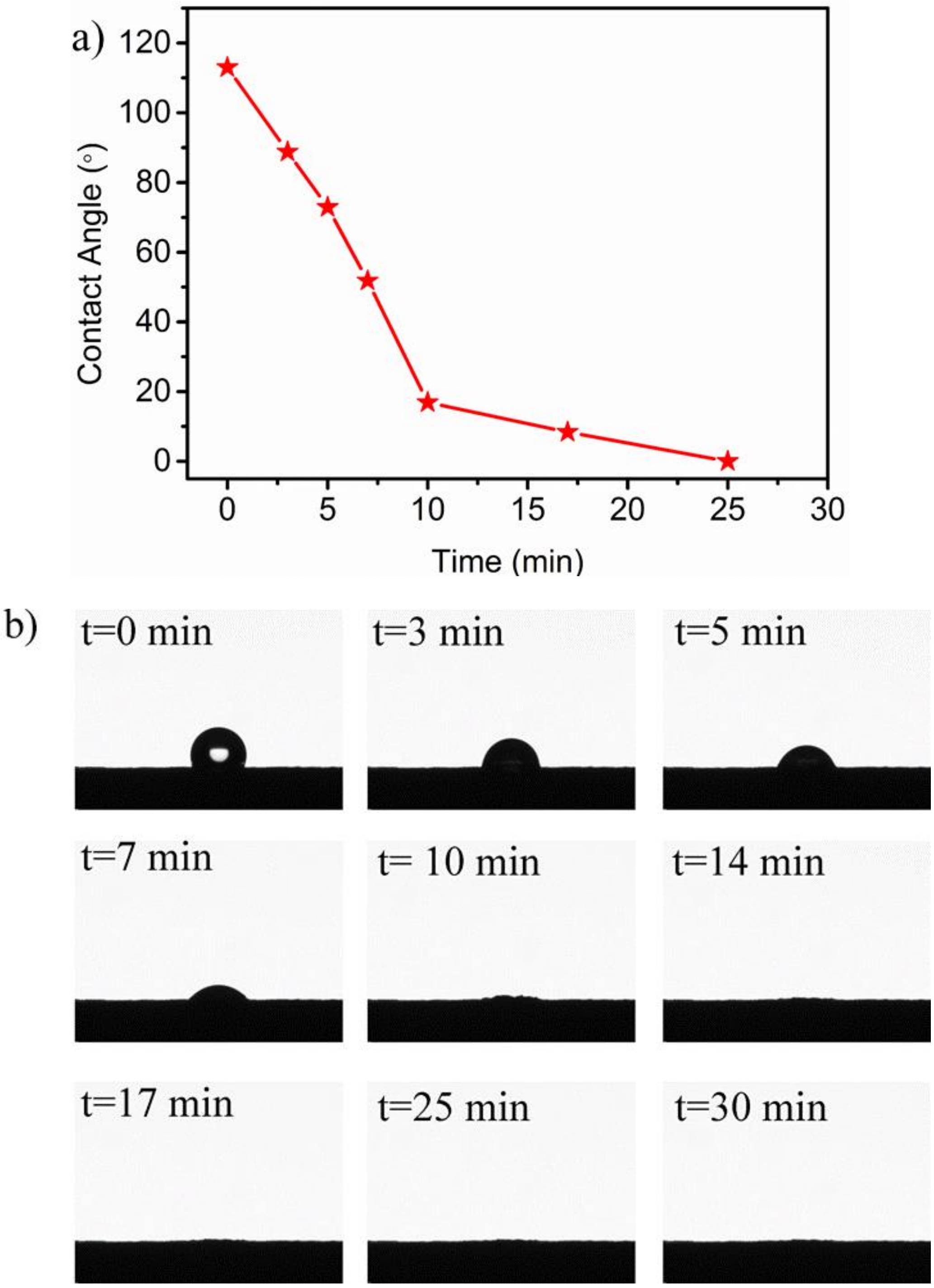

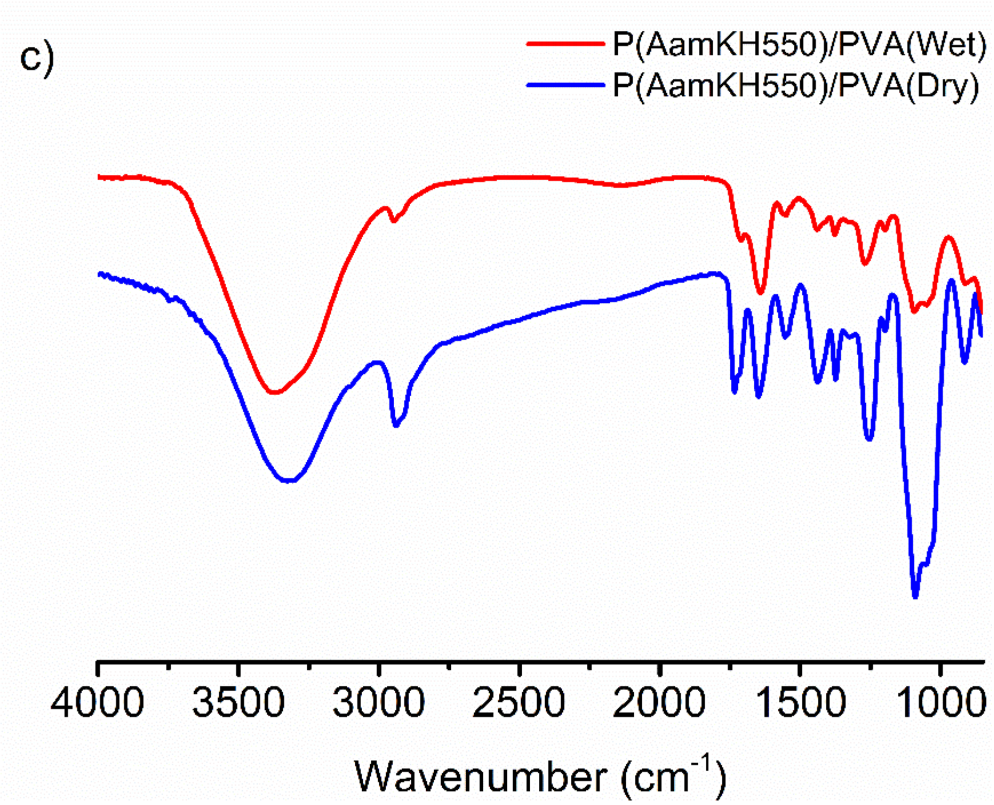

Figure S9 Water absorbing ability of **P(AamKH550)/PVA**. a) The contact angle measurements of **P(AamKH550)/PVA** strip. b) Images of contact angle measurements at 0 min, 3 min, 5 min, 7 min, 10 min, 14 min, 17 min, 25 min, 30 min. c) IR spectra of dry and wet **P(AamKH550)/PVA**.

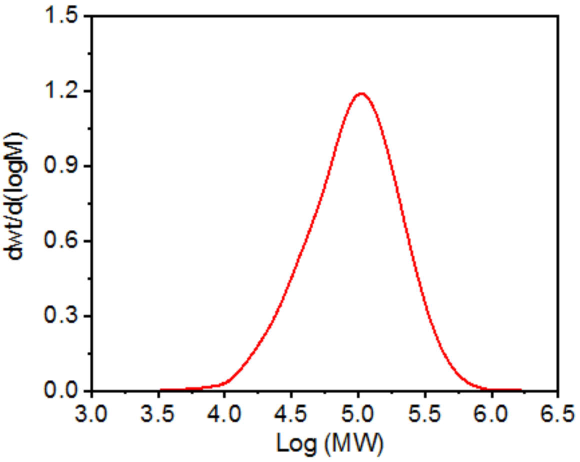

Figure S10 GPC trace of **P(AamKH550)**.

3. Supplementary Tables

Table S1. Data of mechanical tensile property of **P(AamKH550)/PVA**.

	P(AamKH550)/PVA
Young's modulus (MPa)	1187.68 ± 67.67
Tensile strength (MPa)	17.49 ± 0.56
Breaking strain (%)	8.92 ± 0.19

Table S2. Data of mechanical bending property of **P(AamKH550)/PVA**.

	P(AamKH550)/PVA
Young's modulus (MPa)	187.39±15.27
Bending strength (MPa)	29.67±0.49

Table S3. The effect of healing humidity of **P(AamKH550)/PVA**.

Healing humidity	Healing temperature (°C)	Healing time (h)	Young's modulus (MPa)	Tensile strength (MPa)	Breaking strain (mm/mm)
Pristine	-	-	1254.09	16.85	9.20
23%	25	24	1258.05	12.74	6.20
43%	25	24	934.33	6.09	3.76
75%	25	24	853.50	3.69	2.40

Table S4. The effect of healing temperature of **P(AamKH550)/PVA**.

Healing temperature (°C)	Healing humidity (%)	Healing time (h)	Young's modulus (MPa)	Tensile strength (MPa)	Breaking strain (mm/mm)
Pristine	-	-	1233.26	17.54	8.79
25	23	24	1258.05	12.74	6.20
50	23	24	1299.31	13.14	5.35
60	23	24	1169.45	14.51	6.71
70	23	24	1110.74	17.41	8.60

Table S5. The effect of healing time of **P(AamKH550)/PVA**.

Healing time (h)	Healing humidity (%)	Healing temperature (°C)	Young's modulus (MPa)	Tensile strength (MPa)	Breaking strain (mm/mm)
Pristine	-	-	1153.80	17.76	8.79
1	23	25	924.91	1.42	0.87
3	23	25	968.08	6.41	2.84
6	23	25	1040.46	7.97	4.40
12	23	25	1055.83	10.51	5.45
24	23	25	1258.05	12.74	6.20

Table S6. The comparison data of mechanical properties and healing properties of **P(AamKH550)/PVA**, **P(AamKH550-Aam)/PVA**, **PAam/PVA**.

Polymer	Young's modulus (MPa)	Tensile strength pristine (MPa)	Breaking strain (mm/mm)	Tensile strength healed (MPa)
**P(AamKH550)/PVA**	1109.57	17.48	8.88	17.95
**P(AamKH550-Aam)/PVA**	595.62	12.15	28.05	11.74
**PAam/PVA**	7.80	1.13	87.88	0.96

Table S7. The recycling properties of **P(AamKH550)/PVA**.

Cycle number	Young's modulus (MPa)	Tensile strength (MPa)	Breaking strain (mm/mm)
1	1068.17	18.49	9.16
2	1071.03	18.00	8.85
3	1123.36	16.98	7.51

4	1260.07	17.29	7.95

Table S8. The molecular weight and PDI of **P(AamKH550)**.

Mn	Mw	PDI
64250	123657	1.92

References

1. Timofeev, E.N.; Kochetkova, S.V.; Mirzabekov, A.D.; Florentiev, V.L. Regioselective immobilization of short oligonucleotides to acrylic copolymer gels. *Nucleic Acids Res.* **1996**, *24*, 3142–3148.
2. Caprasse, F.; Leroy, D.; Martinot, L.; Lambert, S.; Pirard, J.P.; Guillaume, J.; Jérôme, R. New silica based polymeric systems designed for the solid–liquid extraction of uranyl ions. *J. Mater. Chem.* **2002**, *12*, 137–142.
3. Bai, R.; Yang, J.; Morelle, X.P.; Suo, Z. Flaw‐insensitive hydrogels under static and cyclic loads. *Macromol. Rapid Commun.* **2019**, *40*, 1800883.
